# Prognostic relevance of demographic factors in cardiac magnetic resonance-proven acute myocarditis: A cohort study

**DOI:** 10.3389/fcvm.2022.1037837

**Published:** 2022-10-13

**Authors:** Antonio Cannata, Prashan Bhatti, Roman Roy, Mohammad Al-Agil, Allen Daniel, Emma Ferone, Antonio Jordan, Barbara Cassimon, Susie Bradwell, Abdullah Khawaja, Matthew Sadler, Aamir Shamsi, Josef Huntington, Alexander Birkinshaw, Irfan Rind, Stefania Rosmini, Susan Piper, Daniel Sado, Mauro Giacca, Ajay M. Shah, Theresa McDonagh, Paul A. Scott, Daniel I. Bromage

**Affiliations:** ^1^British Heart Foundation Centre of Research Excellence, School of Cardiovascular Medicine and Sciences, King’s College London, London, United Kingdom; ^2^King’s College Hospital NHS Foundation Trust, London, United Kingdom

**Keywords:** myocarditis, presentation, sex, ethnicity, outcomes, cardiac magnetic resonance (CMR)

## Abstract

**Aim:**

Acute myocarditis (AM) is a heterogeneous condition with variable estimates of survival. Contemporary criteria for the diagnosis of clinically suspected AM enable non-invasive assessment, resulting in greater sensitivity and more representative cohorts. We aimed to describe the demographic characteristics and long-term outcomes of patients with AM diagnosed using non-invasive criteria.

**Methods and results:**

A total of 199 patients with cardiac magnetic resonance (CMR)-confirmed AM were included. The majority (*n* = 130, 65%) were male, and the average age was 39 ± 16 years. Half of the patients were White (*n* = 99, 52%), with the remainder from Black and Minority Ethnic (BAME) groups. The most common clinical presentation was chest pain (*n* = 156, 78%), with smaller numbers presenting with breathlessness (*n* = 25, 13%) and arrhythmias (*n* = 18, 9%). Patients admitted with breathlessness were sicker and more often required inotropes, steroids, and renal replacement therapy (*p* < 0.001, *p* < 0.001, and *p* = 0.01, respectively). Over a median follow-up of 53 (IQR 34–76) months, 11 patients (6%) experienced an adverse outcome, defined as a composite of all-cause mortality, resuscitated cardiac arrest, and appropriate implantable cardioverter defibrillator (ICD) therapy. Patients in the arrhythmia group had a worse prognosis, with a nearly sevenfold risk of adverse events [hazard ratio (HR) 6.97; 95% confidence interval (CI) 1.87–26.00, *p* = 0.004]. Sex and ethnicity were not significantly associated with the outcome.

**Conclusion:**

AM is highly heterogeneous with an overall favourable prognosis. Three-quarters of patients with AM present with chest pain, which is associated with a benign prognosis. AM presenting with life-threatening arrhythmias is associated with a higher risk of adverse events.

## Introduction

Acute myocarditis (AM) is an inflammatory disease of the myocardium occurring most commonly after viral infection, autoimmune disease, or exposure to toxins (1–3). It is characterised by a heterogeneous clinical presentation, ranging from subclinical or minimally symptomatic forms to a life-threatening fulminant presentation with cardiogenic shock or cardiac arrest (4–6).

Early studies of AM mostly included patients with endomyocardial biopsy (EMB)-confirmed AM and suggested a relatively high mortality rate (∼25% at 5 years) (7–11). However, EMB is invasive and therefore reserved for more severe cases, and these studies are likely to have selected more high-risk patients. Recent studies, using cardiovascular magnetic resonance (CMR) in place of EMB to confirm the diagnosis of AM (12, 13), have suggested a more benign clinical course and that clinical presentation can predict prognosis (14–20).

Many of these studies were either small or included small numbers of cases from contributing hospitals, with a consequent risk of selection bias. Furthermore, very few studies report patient ethnicity, and a vast majority of patients in studies where it is reported are White, reflecting local demographics (8, 15, 18). Finally, the impact of sex in a robust, CMR-proven cohort has not been investigated. The objective of this study was to describe the demographic characteristics and outcomes of a large, unselected and ethnically diverse population of patients with AM admitted to a large tertiary Centre in the UK.

## Materials and methods

### Study design

This was a retrospective cohort study. We included all consecutive patients aged ≥18 years admitted to two hospitals within one hospital Trust (King’s College Hospital, London, UK and Princess Royal University Hospital, Orpington, UK), which has a dedicated myocarditis service, between 12th February 2009 and 4th October 2021 with a diagnosis of AM.

The study was conducted under London South-East Research Ethics Committee approval (reference 18/LO/2048) granted to the King’s Electronic Records Research Interface (KERRI) (21). We used an open-source retrieval system for unstructured clinical data (CogStack) to identify patients. The CogStack engine, developed at King’s College London (22), was used with Elasticsearch to process structured and unstructured textual clinical data from various hospital databases. Additional data cleansing was performed using Python in JupyterLab and returned as CSV spreadsheets.

Further unique patients were identified from our local Hospital Episode Statistics (HES) data if they had a discharge diagnosis of AM in the first diagnostic position according to appropriate ICD codes (B33.2 viral carditis, I01.2 acute rheumatic myocarditis, I09.0 rheumatic myocarditis, I40 AM, I41 myocarditis in diseases classified elsewhere, I51.4 myocarditis, unspecified). We also searched the hospital and Intensive Care Unit (ICU) discharge summaries for inpatients discharged alive containing the keywords “myocarditis” or “myopericarditis.” Finally, we searched for patients that died during the study period where the keywords “myocarditis” or “myopericarditis” were given as a cause of death on the death notification. Patients and public were not involved in the design of this study.

Diagnostic criteria for AM were defined according to 2013 ESC-position statement on myocarditis and 2018 Lake Louise Criteria for AM (4, 11, 23, 24), which were independently applied to retrieved data by a minimum of two authors. Patients were included if they presented to the hospital with a consistent clinical presentation and one or more diagnostic criteria based on ECG, cardiac enzymes or cardiac imaging structure and function, in the absence of significant coronary artery disease (CAD) on invasive or non-invasive coronary imaging or low likelihood of CAD (patients <40 years with low clinical suspicion). In addition, all patients had CMR confirmation of AM according to consensus recommendations (24). CMR was considered acceptable if it included cine imaging, late-gadolinium enhancement imaging, and parametric mapping of myocardial T1 and T2. Patients with suspected/confirmed COVID-19 or vaccine-related AM were excluded. Patients with cardiac sarcoidosis were also excluded. All patients underwent a history and physical examination. Laboratory parameters, echocardiography and CMR findings were recorded.

### Clinical presentation, demographic characteristics, and outcomes

We categorised patients into three groups based on their main clinical presentation: (1) chest pain; (2) breathlessness; and (3) arrhythmia. Chest pain presentations were defined as acute chest pain (either ischaemic or pericarditic-sounding) in the absence of CAD and significant left ventricular systolic dysfunction (LVSD). Breathlessness presentations were defined as new onset or progressive HF syndrome. Life-threatening arrhythmias were defined as advanced atrioventricular block, sustained ventricular arrhythmias, or aborted sudden cardiac death. We also categorised patents according to their self-reported ethnicity, and sex. Main clinical presentation was defined by interrogation of the medical record by two authors (DB and AC). Disagreements were resolved by a third author (PS). For each patient, data on baseline co-morbidities and cardiovascular risk factors were obtained using a mix of CogStack retrieval, which was manually validated, and manual searches of the electronic patient record. The primary outcome was a composite of all-cause mortality, resuscitated cardiac arrest, and appropriate implantable cardioverter defibrillator (ICD) therapy following hospital discharge. Appropriate ICD therapy was defined as an appropriate shock for life-threatening arrhythmias.

### Statistical analysis

The results were reported in line with the “Strengthening the Reporting of Observational Studies in Epidemiology” (STROBE) guidelines (Supplementary Table 1) (25). Continuous variables were expressed as mean and standard deviation, or median and interquartile range (IQR), where appropriate. Categorical variables were expressed as counts and percentage. Comparisons between groups were made by the analysis of variance (ANOVA) test on continuous variables or the Student’s *t*-test, or by the non-parametric Mann–Whitney test when appropriate. The Chi-square test or the Fisher’s exact tests were calculated for discrete variables.

Survival curves for the primary outcome were estimated and compared between groups by means of the log-rank test. Univariable Cox regression models were performed to obtain hazard ratios for adverse events in the study population. Given the low number of events in the population, multivariable analysis was not performed to avoid overfitting. *p* < 0.05 was considered significant. All analyses were performed with IBM SPSS Statistics Version 28.0 (IBM Corp., Armonk, NY, USA) and R (R Foundation for Statistical Computing, Vienna, Austria).

## Results

### Derivation of the cohort

Screening hospital and ICU discharge summaries for keywords “myocarditis” or “myopericarditis” revealed 683 unique patients. Three additional patients were identified by screening death notifications for the same keywords. A further 152 unique patients were identified by searching for admissions with ICD-10 codes corresponding to myocarditis. Discharge summaries and patient records were screened for eligibility and 207 cases were excluded due to not being a discharge diagnosis or death notification of AM. A further eight patients were excluded as they were younger than 18 years old at time of admission. Next, data on diagnostic criteria were extracted. 196 patients had insufficient evidence of AM, CAD was not sufficiently excluded in 18, and an alternative diagnosis was evident in 161. A further 49 patients had clinically suspected AM that was not proven by CMR or EMB. Finally, 199 patients with CMR-proven AM were included in our cohort. Only one patient underwent EMB.

### Baseline characteristics

A total of 199 patients with CMR-proven AM were included in the study (Table 1). The majority (*n* = 130, 65%) were male and the average age was 39 ± 16 years. The study population was ethnically diverse. While most patients were White (*n* = 99, 52%), Black, African, Caribbean, or Black British (*n* = 61, 32%), Asian (*n* = 8, 4%), and “other” (*n* = 24, 12%) ethnicities were also represented. The most common clinical presentation was with chest pain (*n* = 149, 75%), with smaller numbers presenting with breathlessness (*n* = 32, 16%) and arrhythmias (*n* = 18, 9%). Fewer than half of patients (*n* = 80, 41%) experienced prodromal symptoms, which included flu-like symptoms in 26% (*n* = 51). Only a small number of patients had a pre-existing autoimmune disorder (*n* = 24, 12%) and the most common cardiac co-morbidity was hypertension (*n* = 26, 13%). Among those with arrhythmic presentation, 14 patients had sustained ventricular tachycardia or ventricular fibrillation.

**TABLE 1:** Characteristics of study patients.

Baseline characteristics		
	*n* patients	199
	Male sex, *n* (%)	130 (65%)
	Age at admission, years	39.2 ± 16.3
Ethnicity	White, *n* (%)	99 (52%)
	Black, African, Caribbean, or Black British, *n* (%)	61 (32%)
	Asian, *n* (%)	8 (4.2%)
	Other, *n* (%)	24 (12%)
History	Any prodromal symptoms, *n* (%)	80 (41%)
	Flu-like symptoms, *n* (%)	51 (26%)
Clinical presentation	Chest pain	149 (75%)
	Breathlessness	32 (16%)
	Arrhythmia	18 (9.0%)
Co-morbidities	Autoimmune disorders, *n* (%)	24 (12%)
	Previous myocarditis, *n* (%)	7 (3.6%)
	Hypertension, *n* (%)	26 (13%)
	Dyslipidaemia, *n* (%)	11 (5.6%)
	Diabetes, *n* (%)	12 (6.1%)
	CKD, *n* (%)	8 (4.1%)
	PAD, *n* (%)	1 (0.5%)
	Previous MI, *n* (%)	1 (0.5%)
	Alcohol, *n* (%)	4 (2.0%)
Baseline medication	RAASi, *n* (%)	14 (7.3%)
	Beta blocker, *n* (%)	15 (7.8%)
	MRA, *n* (%)	0 (0%)
	Diuretics, *n* (%)	5 (2.6%)
	Immunosuppressant, *n* (%)	19 (9.9%)
	Statin, *n* (%)	19 (9.9%)
	Aspirin, *n* (%)	17 (8.9%)
Admission observations	Temperature, °C	37.04 ± 0.80
	Fever > 37.5, *n* (%)	36 (23%)
	Systolic blood pressure, mmHg	121 ± 19
	Heart rate, bpm	83 ± 23
Presenting ECG	Normal, *n* (%)	50 (26%)
	Sinus rhythm, *n* (%)	173 (92%)
	ST elevation, *n* (%)	48 (26%)
	Other repolarisation abnormalities, *n* (%)	72 (39%)
	LBBB, *n* (%)	9 (5%)
	QRS duration, ms	95 ± 18
	QT_c_	396 ± 53
	Q waves	12 (6.9%)
Bloods	Creatinine, mg/dl	98 ± 76
	eGFR, ml/min/m^2^	77 ± 22
	Urea, mmol/L	6.3 ± 6.0
	Sodium, mEq/L	137.8 ± 4.2
	Potassium, mEq/L	4.32 ± 0.60
	Haemoglobin, g/dl	135 ± 21
	CRP, U/L	65 ± 85
	White cell count, 10^9^/L	148 (79%)
	Neutrophils, 10^9^/L	9.8 ± 4.3
	Lymphocytes, 10^9^/L	7.3 ± 4.2
	Monocytes, 10^9^/L	1.73 ± 0.85
	Basophils, 10^9^/L	0.61 ± 0.30
	Eosinophils, 10^9^/L	0.04 ± 0.05
	ESR, mm/h	0.12 ± 0.18
	TSH, mIU/L	37 ± 38
	Free T4, pmol/L	2.28 ± 2.48
	Peak troponin, ×ULN	771 ± 1,570
	NT-proBNP, pg/ml	1,272 ± 5,734
Echo	LVEDD, mm	48.5 ± 6.4
	LVEDV, ml	112 ± 35
	IVSDd, mm	10.01 ± 2.44
	No LVSD, *n* (%)	108 (63%)
	Mild LVSD, *n* (%)	20 (12%)
	Moderate LVSD, *n* (%)	23 (13%)
	Severe LVSD, *n* (%)	20 (12%)
	LVEF, %	51 ± 11
CMR	LVEDV indexed, ml/m^2^	86 ± 17
	LV mass indexed, g/m^2^	57 ± 15
	LVEF, %	57 ± 10
	RVEDV indexed, ml/m^2^	88 ± 27
	RVEF, %	57 ± 7
**In-hospital management**		
Place of care	Cardiology, *n* (%)	132 (68%)
	General medicine, *n* (%)	46 (24%)
	Other wards, *n* (%)	16 (8.2%)
Advanced therapies	Inotropes, *n* (%)	18 (9.4%)
	Intravenous immunoglobulin, *n* (%)	2 (1.0%)
	Steroids, *n* (%)	14 (7.3%)
	Renal replacement therapy, *n* (%)	12 (6.2%)
Discharge medication	Aspirin, *n* (%)	72 (37%)
	Colchicine, *n* (%)	41 (21%)
	RAASi, *n* (%)	94 (48%)
	Beta blocker, *n* (%)	98 (51%)
	MRA, *n* (%)	11 (5.7%)
	Diuretics, *n* (%)	21 (11%)
	Statin	31 (16%)
	Amiodarone, *n* (%)	3 (1.5%)
	Immunosuppressant, *n* (%)	28 (14%)

CKD, chronic kidney disease; PAD, peripheral arterial disease; MI, myocardial infarction; RAASi, renin angiotensin aldosterone system inhibitors; MRA, mineralocorticoid receptor antagonist; LBBB, left bundle branch block; eGFR, estimate glomerular filtration rate; CRP, C-reactive protein; ESR, erythrocyte sedimentation rate; TSH, thyroid stimulating hormone; NT-proBNP, N-terminal pro-brain natriuretic peptide; LVEDD, left ventricular end-diastolic dimension; LVEDV, left ventricular end diastolic volume; IVSDd, Interventricular septal diameter in diastole; LVSD, left ventricular systolic dysfunction; LVEF, left ventricular ejection fraction; LV, left ventricle; RVEDV, right ventricular end diastolic volume; RVEF, right ventricular ejection fraction.

At admission, all patients underwent ECG, blood testing and CMR as a minimum. Most patients had an abnormal ECG at presentation (*n* = 149, 74%), predominantly consisting of repolarisation abnormalities (*n* = 72, 39%) with a smaller proportion demonstrating ST elevation (*n* = 48, 26%). Overall, renal function was mildly impaired (eGFR 77 ± 22 ml/min/m^2^) with elevated inflammatory markers (CRP 65 ± 85 U/L). Peak troponin was elevated at 771 times the upper limit of normal (×ULN). Of patients that had an echo at baseline (*n* = 173, 87%), two thirds presented with normal left ventricular ejection fraction (LVEF).

Overall, most patients (*n* = 132, 68%) were managed in a cardiology ward. Only a small minority were treated with advanced therapies, including inotropes (*n* = 18, 9%), and renal replacement therapy (RRT) (*n* = 12, 6%). Immunosuppression was started in 16 patients (8%). Specifically, 2 patients (1%) received intravenous immunoglobulin (IVIG) and 14 patients received steroids (7%). At discharge, approximately half of patients were on renin-angiotensin-aldosterone system (RAAS) inhibitors. Cardiac devices were present in 21 patients (8%).

### Clinical presentation

Baseline characteristics according to clinical presentation are summarised in Table 2. Patients presenting with chest pain were younger and more frequently male compared to other presentations, while ethnicity was equally distributed. Co-morbidities and baseline medications were broadly similar between patients with different presentations, although fewer patients with a chest pain presentation had hypertension (*p* = 0.018) and more patients in the breathlessness group were on immunosuppression (*p* = 0.002). Admission observations were similar between groups, though patients with a breathlessness presentation had a significantly higher heart rate than chest pain or arrhythmia presentations (*p* < 0.001).

**TABLE 2:** Characteristics of study patients according to clinical presentation.

Baseline characteristics		Chest pain	Breathlessness	Arrhythmia	*P*-value
	*n* patients	156 (78%)	25 (13%)	18 (9%)	–
	Male sex, *n* (%)	114 (73%)	5 (20%)	11 (61%)	<0.001
	Age at admission, years	35.8 ± 14.5	51.0 ± 18.8	52.6 ± 14.0	<0.001
Ethnicity	White, *n* (%)	99 (52%)	76 (50%)	14 (58%)	0.983
	Black, African, Caribbean, or Black British, *n* (%)	61 (32%)	49 (32%)	7 (29%)	
	Asian, *n* (%)	8 (4.2%)	6 (4.0%)	1 (4.2%)	
	Other, *n* (%)	24 (12%)	20 (13%)	2 (8.3%)	
History	Any prodromal symptoms, *n* (%)	68 (44%)	8 (32%)	4 (22%)	0.129
	Flu-like symptoms, *n* (%)	45 (29%)	3 (12%)	3 (17%)	0.136
Co-morbidities	Autoimmune disorders, *n* (%)	15 (9.7%)	6 (24%)	3 (17%)	0.092
	Previous myocarditis, *n* (%)	5 (3.2%)	1 (4.0%)	1 (5.6%)	0.622
	Hypertension, *n* (%)	15 (9.7%)	7 (28%)	4 (22%)	0.018
	Dyslipidaemia, *n* (%)	7 (4.5%)	1 (4.0%)	3 (17%)	0.120
	Diabetes, *n* (%)	7 (4.5%)	3 (12%)	2 (11%)	0.130
	CKD, *n* (%)	3 (1.9%)	4 (16%)	1 (5.6%)	0.007
	PAD, *n* (%)	1 (0.6%)	0 (0%)	0 (0%)	>0.999
	Previous MI, *n* (%)	1 (0.6%)	0 (0%)	0 (0%)	>0.999
	Alcohol, *n* (%)	1 (0.6%)	0 (0%)	3 (17%)	0.004
Baseline medication	RAASi, *n* (%)	11 (7.4%)	1 (4.0%)	2 (11%)	0.777
	Beta blocker, *n* (%)	9 (6.0%)	3 (12%)	3 (17%)	0.129
	MRA, *n* (%)	0 (0%)	0 (0%)	0 (0%)	–
	Diuretics, *n* (%)	2 (1.3%)	2 (8.0%)	1 (5.6%)	0.075
	Immunosuppressant, *n* (%)	9 (6.0%)	7 (28%)	3 (17%)	0.002
	Statin, *n* (%)	11 (7.4%)	2 (8.0%)	6 (33%)	0.007
	Aspirin, *n* (%)	11 (7.4%)	2 (8.0%)	4 (22%)	0.118
Admission observations	Temperature, °C	37.04 ± 0.82	37.13 ± 0.86	36.90 ± 0.45	0.850
	Fever > 37.5, *n* (%)	28 (23%)	7 (37%)	1 (7.1%)	0.152
	Systolic blood pressure, mmHg	121 ± 16	124 ± 31	117 ± 18	0.576
	Heart rate, bpm	80 ± 17	100 ± 26	84 ± 44	<0.001
Presenting ECG	Normal, *n* (%)	39 (26%)	5 (23%)	6 (33%)	0.763
	Sinus rhythm, *n* (%)	141 (95%)	20 (91%)	12 (67%)	0.001
	ST elevation, *n* (%)	45 (31%)	3 (14%)	0 (0%)	0.004
	Other repolarisation abnormalities, *n* (%)	54 (37%)	10 (45%)	8 (44%)	0.65
	LBBB, *n* (%)	4 (3%)	1 (5%)	4 (25%)	0.006
	QRS duration, ms	94 ± 15	91 ± 19	113 ± 32	0.017
	QT_c_	389 ± 47	413 ± 71	432 ± 57	0.005
	Q waves	7 (5.1%)	4 (18%)	1 (7.1%)	0.070
Bloods	Creatinine, umol/L	90 ± 68	127 ± 101	121 ± 91	0.084
	eGFR, ml/min/m^2^	81 ± 18	60 ± 30	64 ± 25	<0.001
	Urea, mmol/L	5.3 ± 3.8	11.8 ± 12.1	7.4 ± 4.5	<0.001
	Sodium, mEq/L	138.2 ± 2.8	136.1 ± 5.9	137.3 ± 9.0	0.029
	Potassium, mEq/L	4.23 ± 0.48	4.70 ± 0.82	4.58 ± 0.86	0.006
	Haemoglobin, g/dl	138 ± 17	117 ± 27	136 ± 29	<0.001
	CRP, U/L	60 ± 81	114 ± 100	41 ± 82	<0.001
	White cell count, 10^9^/L	119 (82%)	21 (88%)	8 (44%)	0.002
	Neutrophils, 10^9^/L	9.2 ± 3.6	12.4 ± 6.4	11.8 ± 4.9	0.007
	Lymphocytes, 10^9^/L	6.7 ± 3.4	10.2 ± 6.3	8.7 ± 4.9	0.014
	Monocytes, 10^9^/L	1.72 ± 0.68	1.35 ± 0.97	2.32 ± 1.49	0.006
	Basophils, 10^9^/L	0.61 ± 0.29	0.56 ± 0.35	0.65 ± 0.30	0.568
	Eosinophils, 10^9^/L	0.04 ± 0.04	0.05 ± 0.07	0.04 ± 0.04	0.501
	ESR, mm/h	0.12 ± 0.12	0.16 ± 0.38	0.09 ± 0.14	0.042
	TSH, mIU/L	36 ± 37	36 ± 32	51 ± 57	0.789
	Free T4, pmol/L	2.22 ± 2.62	2.76 ± 2.91	1.99 ± 1.24	0.820
	Peak troponin, ×ULN	858 ± 1,702	301 ± 491	694 ± 1,280	0.020
	NT-proBNP, pg/ml	471 ± 2,936	3,897 ± 10,726	3,162 ± 9,000	0.277
Echo	LVEDD, mm	47.8 ± 5.7	50.0 ± 8.3	52.7 ± 6.9	0.020
	LVEDV, ml	108 ± 30	119 ± 52	130 ± 36	0.074
	IVSDd, mm	10.10 ± 2.40	9.91 ± 2.71	9.39 ± 2.44	0.449
	No LVSD, *n* (%)	95 (72%)	5 (22%)	8 (50%)	<0.001
	Mild LVSD, *n* (%)	15 (11%)	3 (13%)	2 (12%)	
	Moderate LVSD, *n* (%)	14 (11%)	6 (26%)	3 (19%)	
	Severe LVSD, *n* (%)	8 (6.1%)	9 (39%)	3 (19%)	
	LVEF, %	53 ± 9	40 ± 13	45 ± 12	<0.001
CMR	LVEDV indexed, ml/m^2^	86 ± 15	80 ± 24	98 ± 31	0.423
	LV mass indexed, g/m^2^	57 ± 15	59 ± 13	70 ± 20	0.166
	LVEF, %	58 ± 9	51 ± 15	55 ± 9	0.017
	RVEDV indexed, ml/m^2^	88 ± 18	70 ± 26	143 ± 100	0.124
	RVEF, %	56 ± 8	61 ± 8	55 ± 4	0.213
**In-hospital management**					
Place of care	Cardiology, *n* (%)	116 (76%)	4 (17%)	12 (71%)	<0.001
	General medicine, *n* (%)	33 (22%)	10 (42%)	3 (18%)	
	Other wards, *n* (%)	4 (2.6%)	10 (42%)	2 (12%)	
Advanced therapies	Inotropes, *n* (%)	8 (5.3%)	8 (33%)	2 (11%)	<0.001
	Intravenous immunoglobulin, *n* (%)	2 (1.3%)	0 (0%)	0 (0%)	>0.999
	Steroids, *n* (%)	6 (4.0%)	7 (29%)	1 (5.6%)	<0.001
	Renal replacement therapy, *n* (%)	6 (4.0%)	5 (21%)	1 (5.6%)	0.010
Discharge medication	Aspirin, *n* (%)	58 (38%)	8 (32%)	6 (33%)	0.779
	Colchicine, *n* (%)	40 (26%)	1 (4.0%)	0 (0%)	<0.001
	RAASi, *n* (%)	66 (44%)	17 (68%)	11 (61%)	0.042
	Beta blocker, *n* (%)	65 (43%)	18 (72%)	15 (83%)	<0.001
	MRA, *n* (%)	4 (2.7%)	6 (24%)	1 (5.6%)	<0.001
	Diuretics, *n* (%)	10 (6.6%)	9 (36%)	2 (11%)	<0.001
	Statin	18 (12%)	5 (21%)	8 (44%)	0.003
	Amiodarone, *n* (%)	0 (0%)	2 (8.0%)	1 (5.6%)	0.010
	Immunosuppressant, *n* (%)	14 (9.3%)	11 (44%)	3 (17%)	<0.001

CKD, chronic kidney disease; PAD, peripheral arterial disease; MI, myocardial infarction; RAASi, renin angiotensin aldosterone system inhibitors; MRA, mineralocorticoid receptor antagonist; LBBB, left bundle branch block; eGFR, estimate glomerular filtration rate; CRP, C-reactive protein; ESR, erythrocyte sedimentation rate; TSH, thyroid stimulating hormone; ULN, upper limit of normal; NT-proBNP, N-terminal pro-brain natriuretic peptide; LVEDD, left ventricular end-diastolic dimension; LVEDV, left ventricular end diastolic volume; IVSDd, Interventricular septal diameter in diastole; LVSD, left ventricular systolic dysfunction; LVEF, left ventricular ejection fraction; LV, left ventricle; RVEDV, right ventricular end diastolic volume; RVEF, right ventricular ejection fraction.

Patients with a chest pain presentation were more likely to have ST elevation than other presentations (31% compared to 14% with breathlessness and none with arrhythmia, *p* = 0.004), while patients in the arrhythmia group were more likely to have LBBB (*p* = 0.006). Patients with breathlessness had worse renal function, lower Hb and higher inflammatory markers than other presentations (*p* < 0.001 for all). Peak troponin was highest in the chest pain group and lowest in those presenting with breathlessness (*p* = 0.02). Mean LVEF was higher in the chest pain group compared to breathlessness and arrhythmia patients (53 ± 9 vs. 40 ± 13 vs. 45 ± 12%, respectively, *p* < 0.001). A total of 78% of patients presenting with breathlessness had LVSD, and more often had moderate or severe LVSD compared to those with chest pain or arrhythmias at presentation (65 vs. 17 vs. 38%, respectively, *p* < 0.001).

In-hospital management was significantly different between groups. Less than one fifth of patients admitted with breathlessness were managed on a cardiology ward compared to three quarters for other presentations (17% for breathlessness vs. 76% for chest pain vs. 71% for arrhythmia, *p* < 0.001). With the exception of IVIG, advanced therapies were more commonly used in breathlessness presentations (inotropes *p* < 0.001, steroids *p* < 0.001, RRT *p* = 0.01). Furthermore, patients with a breathlessness presentation were more likely to be treated with RAAS inhibitors (*p* = 0.042) as well as mineralocorticoid receptor antagonists (MRA, *p* < 0.001), diuretics (*p* < 0.001) and/or immunosuppression (*p* < 0.001). Arrhythmia patients were more likely to be treated with beta blockers (*p* < 0.001) or statins (*p* = 0.003), while patients presenting with chest pain were more likely to be treated with colchicine (*p* < 0.001).

### Ethnicity differences in acute myocarditis

Ethnic differences among patients presenting with AM are presented in Table 3. No differences in age, sex, clinical presentation, comorbidities, or baseline treatment were evident. Compared to White patients, Black and Minority Ethnic (BAME) patients had more repolarisation abnormalities on ECG (47 vs. 32%, *p* = 0.04) and a more pronounced leucocytosis (neutrophils 10.3 ± 4.2 vs. 9.2 ± 4.1 × 10^9^/L, *p* = 0.025; lymphocytes 7.8 ± 4.1 vs. 6.7 ± 4.0 × 10^9^/L, *p* = 0.018). BAME and White patients had similar rates of moderate or severe left ventricular (LV) systolic dysfunction, measured with echo (23 vs. 27%, *p* = 0.47), and no differences on CMR. Inpatient and discharge medications were also indistinct between groups.

**TABLE 3:** Characteristics of study patients according to ethnicity.

Baseline characteristics		BAME	White	*P*-value
	*n* patients	93	99	–
	Male sex, *n* (%)	65 (70%)	59 (60%)	0.136
	Age at admission, years	38.7 ± 14.6	39.2 ± 17.8	0.742
History	Any prodromal symptoms, *n* (%)	43 (47%)	34 (35%)	0.091
	Flu-like symptoms, *n* (%)	26 (28%)	23 (23%)	0.451
Clinical presentation	Chest pain	66 (71%)	78 (79%)	0.293
	Breathlessness	19 (20%)	12 (12%)	
	Arrhythmia	8 (8.6%)	9 (9.1%)	
Co-morbidities	Autoimmune disorders, *n* (%)	10 (11%)	14 (14%)	0.479
	Previous myocarditis, *n* (%)	4 (4.3%)	3 (3.1%)	0.714
	Hypertension, *n* (%)	11 (12%)	13 (13%)	0.786
	Dyslipidaemia, *n* (%)	7 (7.6%)	4 (4.1%)	0.298
	Diabetes, *n* (%)	6 (6.5%)	6 (6.1%)	0.910
	CKD, *n* (%)	4 (4.3%)	3 (3.1%)	0.714
	PAD, *n* (%)	1 (1.1%)	0 (0%)	0.484
	Previous MI, *n* (%)	1 (1.1%)	0 (0%)	0.484
	Alcohol, *n* (%)	2 (2.2%)	2 (2.0%)	>0.999
Baseline medication	RAASi, *n* (%)	6 (6.7%)	7 (7.3%)	0.867
	Beta blocker, *n* (%)	7 (7.8%)	8 (8.3%)	0.889
	MRA, *n* (%)	0 (0%)	0 (0%)	–
	Diuretics, *n* (%)	0 (0%)	5 (5.2%)	0.060
	Immunosuppressant, *n* (%)	8 (8.9%)	11 (11%)	0.563
	Statin, *n* (%)	9 (10%)	9 (9.4%)	0.885
	Aspirin, *n* (%)	8 (8.9%)	9 (9.4%)	0.908
Admission observations	Temperature, °C	37.16 ± 0.87	36.91 ± 0.69	0.067
	Fever > 37.5, *n* (%)	22 (27%)	12 (17%)	0.130
	Systolic blood pressure, mmHg	120 ± 20	122 ± 18	0.225
	Heart rate, bpm	86 ± 26	80 ± 19	0.071
Presenting ECG	Normal, *n* (%)	22 (24%)	25 (27%)	0.706
	Sinus rhythm, *n* (%)	83 (93%)	84 (90%)	0.472
	ST elevation, *n* (%)	21 (24%)	26 (28%)	0.474
	Other repolarisation abnormalities, *n* (%)	42 (47%)	29 (32%)	0.04
	LBBB, *n* (%)	3 (4%)	6 (7%)	0.49
	QRS duration, ms	94 ± 17	96 ± 20	0.764
	QT_c_	392 ± 54	399 ± 52	0.450
	Q waves	7 (8.8%)	5 (5.7%)	0.453
Bloods	Creatinine, mg/dl	107 ± 93	89 ± 56	0.059
	eGFR, ml/min/m^2^	76 ± 22	78 ± 21	0.072
	Urea, mmol/L	5.9 ± 6.2	6.8 ± 5.9	0.004
	Sodium, mEq/L	137.9 ± 3.6	137.8 ± 4.8	0.834
	Potassium, mEq/L	4.29 ± 0.56	4.35 ± 0.59	0.565
	Haemoglobin, g/dl	133 ± 20	138 ± 22	0.053
	CRP, U/L	67 ± 87	57 ± 72	>0.999
	White cell count, 10^9^/L	66 (73%)	76 (84%)	0.051
	Neutrophils, 10^9^/L	9.2 ± 4.1	10.3 ± 4.2	0.025
	Lymphocytes, 10^9^/L	6.7 ± 4.0	7.8 ± 4.1	0.018
	Monocytes, 10^9^/L	1.80 ± 0.86	1.72 ± 0.83	0.427
	Basophils, 10^9^/L	0.57 ± 0.33	0.65 ± 0.25	0.001
	Eosinophils, 10^9^/L	0.04 ± 0.05	0.05 ± 0.04	0.189
	ESR, mm/h	0.13 ± 0.13	0.12 ± 0.22	0.140
	TSH, mIU/L	41 ± 39	34 ± 38	0.390
	Free T4, pmol/L	2.12 ± 1.88	2.42 ± 3.00	0.820
	Peak troponin, ×ULN	916 ± 2,087	643 ± 898	0.351
	NT-proBNP, pg/ml	863 ± 4,471	1,691 ± 6,780	0.263
Echo	LVEDD, mm	47.2 ± 6.4	49.7 ± 6.2	0.018
	LVEDV, ml	107 ± 33	116 ± 37	0.231
	IVSDd, mm	10.46 ± 2.62	9.53 ± 2.17	0.016
	No LVSD, *n* (%)	52 (62%)	53 (66%)	0.348
	Mild LVSD, *n* (%)	9 (11%)	9 (11%)	
	Moderate LVSD, *n* (%)	15 (18%)	7 (8.8%)	
	Severe LVSD, *n* (%)	8 (9.5%)	11 (14%)	
	LVEF, %	51 ± 10	51 ± 12	0.707
CMR	LVEDV indexed, ml/m^2^	84 ± 18	88 ± 17	0.132
	LV mass indexed, g/m^2^	60 ± 18	56 ± 12	0.557
	LVEF, %	58 ± 9	57 ± 11	0.341
	RVEDV indexed, ml/m^2^	86 ± 20	90 ± 32	0.468
	RVEF, %	57 ± 9	56 ± 6	0.546
**In-hospital management**				
Place of care	Cardiology, *n* (%)	60 (67%)	68 (70%)	0.256
	General medicine, *n* (%)	25 (28%)	19 (20%)	
	Other wards, *n* (%)	5 (5.6%)	10 (10%)	
Advanced therapies	Inotropes, *n* (%)	9 (9.9%)	9 (9.5%)	0.924
	Intravenous immunoglobulin, *n* (%)	1 (1.1%)	1 (1.1%)	>0.999
	Steroids, *n* (%)	4 (4.4%)	9 (9.5%)	0.175
	Renal replacement therapy, *n* (%)	5 (5.5%)	7 (7.4%)	0.603
Discharge medication	Aspirin, *n* (%)	32 (35%)	38 (39%)	0.570
	Colchicine, *n* (%)	21 (23%)	20 (21%)	0.683
	RAASi, *n* (%)	42 (46%)	49 (51%)	0.550
	Beta blocker, *n* (%)	45 (49%)	49 (51%)	0.884
	MRA, *n* (%)	4 (4.4%)	7 (7.2%)	0.421
	Diuretics, *n* (%)	10 (11%)	11 (11%)	0.939
	Statin	12 (13%)	17 (18%)	0.393
	Amiodarone, *n* (%)	0 (0%)	3 (3.1%)	0.247
	Immunosuppressant, *n* (%)	12 (13%)	16 (16%)	0.524

BAME, Black and Minority Ethnic group; CKD, chronic kidney disease; PAD, peripheral arterial disease; MI, myocardial infarction; RAASi, renin angiotensin aldosterone system inhibitors; MRA, mineralocorticoid receptor antagonist; LBBB, left bundle branch block; eGFR, estimate glomerular filtration rate; CRP, C-reactive protein; ESR, erythrocyte sedimentation rate; TSH, thyroid stimulating hormone; ULN, upper limit of normal; NT-proBNP, N-terminal pro-brain natriuretic peptide; LVEDD, left ventricular end-diastolic dimension; LVEDV, left ventricular end diastolic volume; IVSDd, Interventricular septal diameter in diastole; LVSD, left ventricular systolic dysfunction; LVEF, left ventricular ejection fraction; LV, left ventricle; RVEDV, right ventricular end diastolic volume; RVEF, right ventricular ejection fraction.

### Sex differences in acute myocarditis

Baseline characteristics according to sex are summarised in Table 4. Compared to men, women were older (44.5 ± 18.1 vs. 36.4 ± 14.6, *p* = 0.003), while ethnicity was equally distributed. Women were also more likely to have a prior autoimmune disorder (*p* < 0.001), hypertension (*p* = 0.002) and renal insufficiency (eGFR < 60 mL/min/m^2^, *p* = 0.023). More women were on aspirin and/or immunosuppression at the time of presentation (*p* = 0.035 and <0.001, respectively).

**TABLE 4:** Characteristics of study patients according to sex.

Baseline characteristics		Female	Male	*P*-value
	*n* patients	69	130	–
	Age at admission, years	44.5 ± 18.1	36.4 ± 14.6	0.003
Ethnicity	White, *n* (%)	40 (59%)	59 (48%)	0.276
	Black, African, Caribbean, or Black British, *n* (%)	17 (25%)	44 (35%)	
	Asian, *n* (%)	4 (5.9%)	4 (3.2%)	
	Other, *n* (%)	7 (10%)	17 (14%)	
History	Any prodromal symptoms, *n* (%)	23 (33%)	57 (45%)	0.127
	Flu-like symptoms, *n* (%)	14 (20%)	37 (29%)	0.188
Clinical presentation	Chest pain	43 (62%)	106 (82%)	0.001
	Breathlessness	20 (29%)	12 (9.2%)	
	Arrhythmia	6 (8.7%)	12 (9.2%)	
Co-morbidities	Autoimmune disorders, *n* (%)	17 (25%)	7 (5.5%)	<0.001
	Previous myocarditis, *n* (%)	2 (2.9%)	5 (3.9%)	>0.999
	Hypertension, *n* (%)	16 (23%)	10 (7.8%)	0.002
	Dyslipidaemia, *n* (%)	5 (7.2%)	6 (4.7%)	0.521
	Diabetes, *n* (%)	7 (10%)	5 (3.9%)	0.116
	CKD, *n* (%)	6 (8.7%)	2 (1.6%)	0.023
	PAD, *n* (%)	1 (1.4%)	0 (0%)	0.350
	Previous MI, *n* (%)	0 (0%)	1 (0.8%)	>0.999
	Alcohol, *n* (%)	0 (0%)	4 (3.1%)	0.300
Baseline medication	RAASi, *n* (%)	8 (12%)	6 (4.8%)	0.088
	Beta blocker, *n* (%)	9 (13%)	6 (4.8%)	0.038
	MRA, *n* (%)	0 (0%)	0 (0%)	–
	Diuretics, *n* (%)	3 (4.4%)	2 (1.6%)	0.348
	Immunosuppressant, *n* (%)	14 (21%)	5 (4.0%)	<0.001
	Statin, *n* (%)	9 (13%)	10 (8.1%)	0.251
	Aspirin, *n* (%)	10 (15%)	7 (5.6%)	0.035
Admission observations	Temperature, °C	37.16 ± 0.84	36.97 ± 0.76	0.132
	Fever > 37.5, *n* (%)	16 (28%)	20 (20%)	0.247
	Systolic blood pressure, mmHg	124 ± 25	119 ± 14	0.376
	Heart rate, bpm	89 ± 24	80 ± 22	0.003
Presenting ECG	Normal, *n* (%)	19 (30%)	31 (25%)	0.471
	Sinus rhythm, *n* (%)	62 (97%)	111 (90%)	0.078
	ST elevation, *n* (%)	5 (7.8%)	43 (35%)	<0.001
	Other repolarisation abnormalities, *n* (%)	29 (46%)	43 (35%)	0.14
	LBBB, *n* (%)	5 (8%)	4 (4%)	0.28
	QRS duration, ms	95 ± 22	96 ± 16	0.067
	QT_c_	409 ± 62	388 ± 45	0.006
	Q waves	7 (11%)	5 (4.5%)	0.120
Bloods	Creatinine, mg/dl	106 ± 96	93 ± 62	0.019
	eGFR, ml/min/m^2^	69 ± 27	81 ± 17	<0.001
	Urea, mmol/L	8.0 ± 8.6	5.4 ± 3.7	0.023
	Sodium, mEq/L	137.2 ± 4.8	138.2 ± 3.8	0.008
	Potassium, mEq/L	4.34 ± 0.65	4.31 ± 0.57	0.756
	Haemoglobin, g/dl	123 ± 22	142 ± 18	<0.001
	CRP, U/L	70 ± 92	63 ± 82	0.508
	White cell count, 10^9^/L	10.8 ± 4.8	9.3 ± 3.9	0.017
	Neutrophils, 10^9^/L	8.5 ± 4.7	6.7 ± 3.7	0.008
	Lymphocytes, 10^9^/L	1.63 ± 0.82	1.78 ± 0.86	0.207
	Monocytes, 10^9^/L	0.56 ± 0.28	0.63 ± 0.30	0.075
	Basophils, 10^9^/L	0.04 ± 0.05	0.04 ± 0.04	0.519
	Eosinophils, 10^9^/L	0.11 ± 0.24	0.12 ± 0.12	0.7
	ESR, mm/h	43 ± 39	34 ± 38	0.180
	TSH, mIU/L	2.77 ± 3.33	1.93 ± 1.59	0.414
	Free T4, pmol/L	14.70 ± 3.29	15.12 ± 2.89	0.530
	Peak troponin, ×ULN	334 ± 632	1,013 ± 1,860	<0.001
	NT-proBNP, pg/ml	3,019 ± 9,003	190 ± 758	0.004
Echo	LVEDD, mm	46.8 ± 5.7	49.5 ± 6.5	0.003
	LVEDV, ml	100 ± 29	119 ± 36	<0.001
	IVSDd, mm	9.58 ± 2.54	10.25 ± 2.36	0.029
	No LVSD, *n* (%)	37 (61%)	71 (65%)	0.392
	Mild LVSD, *n* (%)	5 (8.2%)	15 (14%)	
	Moderate LVSD, *n* (%)	9 (15%)	14 (13%)	
	Severe LVSD, *n* (%)	10 (16%)	10 (9.1%)	
	LVEF, %	49 ± 12	51 ± 10	0.574
CMR	LVEDV indexed, ml/m^2^	79 ± 17	90 ± 16	0.005
	LV mass indexed, g/m^2^	52 ± 16	61 ± 14	<0.001
	LVEF, %	57 ± 12	57 ± 9	0.913
	RVEDV indexed, ml/m^2^	74 ± 18	96 ± 27	<0.001
	RVEF, %	58 ± 9	56 ± 6	0.083
**In-hospital management**				
Place of care	Cardiology, *n* (%)	32 (47%)	100 (79%)	<0.001
	General Medicine, *n* (%)	22 (32%)	24 (19%)	
	Other wards, *n* (%)	14 (21%)	2 (1.6%)	
Advanced therapies	Inotropes, *n* (%)	14 (21%)	4 (3.2%)	<0.001
	Intravenous immunoglobulin, *n* (%)	1 (1.5%)	1 (0.8%)	>0.999
	Steroids, *n* (%)	12 (18%)	2 (1.6%)	<0.001
	Renal replacement therapy, *n* (%)	9 (13%)	3 (2.3%)	<0.001
Discharge medication	Aspirin, *n* (%)	19 (28%)	53 (42%)	0.052
	Colchicine, *n* (%)	14 (21%)	27 (21%)	0.891
	RAASi, *n* (%)	30 (44%)	64 (51%)	0.375
	Beta blocker, *n* (%)	36 (53%)	62 (49%)	0.620
	MRA, *n* (%)	6 (8.8%)	5 (4.0%)	0.200
	Diuretics, *n* (%)	11 (16%)	10 (7.9%)	0.078
	Statin	12 (18%)	19 (15%)	0.658
	Amiodarone, *n* (%)	2 (2.9%)	1 (0.8%)	0.281
	Immunosuppressant, *n* (%)	20 (29%)	8 (6.3%)	<0.001

BAME, Black and Minority Ethnic group; CKD, chronic kidney disease; PAD, peripheral arterial disease; MI, myocardial infarction; RAASi, renin angiotensin aldosterone system inhibitors; MRA, mineralocorticoid receptor antagonist; LBBB, left bundle branch block; eGFR, estimate glomerular filtration rate; CRP, C-reactive protein; ESR, erythrocyte sedimentation rate; TSH, thyroid stimulating hormone; ULN, upper limit of normal; NT-proBNP, N-terminal pro-brain natriuretic peptide; LVEDD, left ventricular end-diastolic dimension; LVEDV, left ventricular end diastolic volume; IVSDd, Interventricular septal diameter in diastole; LVSD, left ventricular systolic dysfunction; LVEF, left ventricular ejection fraction; LV, left ventricle; RVEDV, right ventricular end diastolic volume; RVEF, right ventricular ejection fraction.

Women were more likely to present with breathlessness compared to men (*p* = 0.001), and were also more tachycardic at admission (89 ± 24 vs. 80 ± 22 bpm, *p* = 0.003). Men were more likely to have a chest pain presentation along with ST elevation (*p* < 0.001 for both).

Women with AM had worse renal function (*p* < 0.001 for eGFR), lower Hb (*p* < 0.001) and higher NT-proBNP (*p* = 0.004) than men. However, men had significantly higher peak troponin (*p* < 0.001). Inflammatory markers were similar between the groups. Mean LVEF was similar between the groups using both echo and CMR, but both modalities indicated more LV dilatation in men (*p* < 0.001 for echo).

In-hospital management was significantly different between groups with higher use of steroids, inotropes and RRT in women (*p* < 0.001 for all). Immunosuppression was also more commonly prescribed for women at discharge (*p* < 0.001), but other treatments were similar.

### Outcomes

Over a median follow-up of 53 (IQR 34–76) months, 11 patients (6%) experienced the primary outcome. Of those, 10 died (5 in the chest pain group, 2 in the breathlessness group and 3 in the arrhythmia group) and 1 patient experienced a successful appropriate ICD shock for monomorphic VT. No patients had resuscitated cardiac arrest.

**FIGURE 1:**
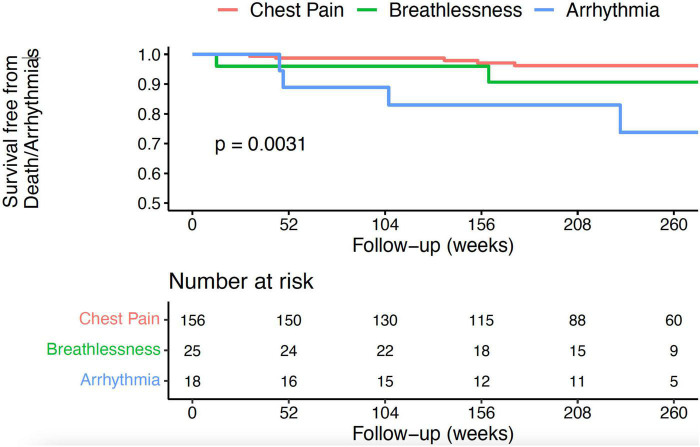
Kaplan–Meier curve for all-cause mortality, resuscitated cardiac arrest, and appropriate implantable cardioverter defibrillator (ICD) therapy following hospital discharge in patients with AM, according to clinical presentation.

**FIGURE 2:**
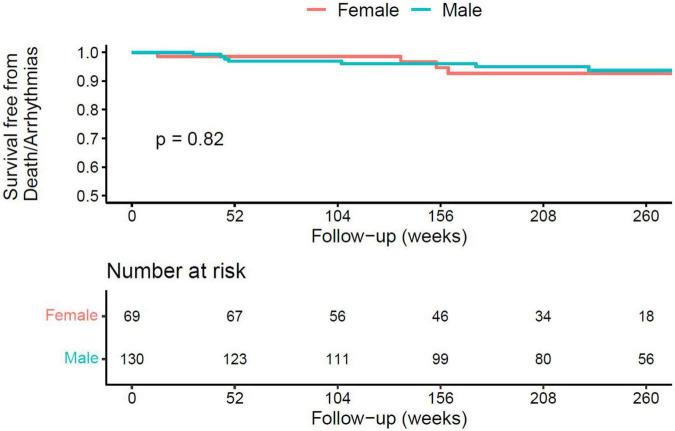
Kaplan–Meier curve for all-cause mortality, resuscitated cardiac arrest, and appropriate implantable cardioverter defibrillator (ICD) therapy following hospital discharge in patients with AM, according to ethnicity.

**FIGURE 3:**
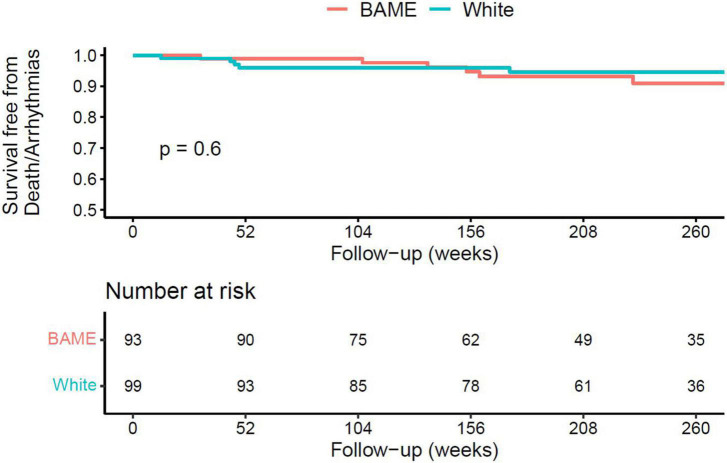
Kaplan–Meier curve for all-cause mortality, resuscitated cardiac arrest, and appropriate implantable cardioverter defibrillator (ICD) therapy following hospital discharge in patients with AM, according to sex.

Overall, 3-year, event-free survival was 96%. Patients in the chest pain group had a more favourable prognosis compared to those presenting with breathlessness or arrhythmia (Figure 1, 3-year event-free survival 98% in the chest pain group, 96% in the breathlessness group, and 89% in the arrhythmia group, *p* = 0.003). Outcomes were similar between ethnicities (Figure 2, 3-year event-free survival 97% in White patients vs. 96% in BAME patients, *p* = 0.6) and sexes (Figure 3, 3-year event-free survival 96% in females vs. 97% in males, *p* = 0.82).

In univariable analyses, the variables most strongly associated with the primary outcome were increasing age (*p* = 0.003), arrhythmia clinical presentations (*p* = 0.004), comorbidities including hypertension, dyslipidaemia and diabetes (all *p* < 0.001), sodium (*p* = 0.003), and LV septal wall thickness (*p* = 0.045) (Table 5). Compared to chest pain presentations, arrhythmia presentations were associated with a nearly sevenfold risk of the primary outcome [hazard ratio (HR) 6.97; 95% confidence interval (CI) 1.87–26.00, *p* = 0.004], whereas breathlessness presentations were not significantly associated with outcome. Sex and ethnicity were not associated with the outcome.

**TABLE 5:** Cox proportional univariable analyses evaluating the association of baseline characteristics with all-cause mortality, resuscitated cardiac arrest, and appropriate implantable cardioverter defibrillator (ICD) therapy following hospital discharge.

Variable		HR	95% CI		*P*-value
	Age at admission (per 10 years)	1.06	1.03	1.1	<0.001
	Male sex	0.87	0.25	2.96	0.82
Ethnicity	White vs. BAME	0.73	0.22	2.39	0.6
History	Any prodromal symptoms	0.14	0.02	1.11	0.06
Clinical presentation	Chest pain	Ref			
	Breathlessness	2.02	0.39	10.4	0.4
	Arrhythmia	6.97	1.87	26	0.004
Co-morbidities	Autoimmune disorders	1.57	0.34	7.25	0.57
	Hypertension	7.91	2.41	25.9	<0.001
	Dyslipidaemia	18.9	5.74	62.1	<0.001
	Diabetes	11.4	3.32	39.2	<0.001
	CKD	2.72	0.35	21.3	0.34
Admission observations	Systolic blood pressure	1	0.97	1.04	0.82
	Heart rate	1	0.98	1.03	0.67
Presenting ECG	Normal	0.28	0.04	2.16	0.22
	Sinus rhythm	0.26	0.07	0.98	0.047
	ST elevation	0.58	0.13	2.71	0.49
	LBBB	1.84	0.72	4.66	0.2
	QT_c_	1	0.99	1.01	0.97
Bloods	Creatinine	1	1	1.01	0.12
	eGFR	0.98	0.96	1	0.11
	Urea	0.98	0.85	1.12	0.74
	Sodium	0.88	0.81	0.96	0.003
	Potassium	1.61	0.72	3.57	0.24
	Haemoglobin	0.98	0.96	1.01	0.16
	CRP	1	0.99	1.01	0.53
	White cell count	1	0.88	1.15	0.96
	Neutrophils	1	0.87	1.15	0.97
	TSH	0.87	0.41	1.82	0.77
	Free T4	1	0.63	1.61	0.99
	Peak troponin	1	1	1	0.63
	NT-proBNP	0.99	0.98	1.01	0.62
Echo	LVEDD	0.99	0.89	1.1	0.85
	LVEDV	1	0.98	1.02	0.89
	IVSDd	1.21	1	1.45	0.045
	No LVSD	Ref	–	–	–
	Mild LVSD	1.23	0.14	11	0.9
	Moderate LVSD	3.99	0.89	17.9	0.07
	Severe LVSD	1.37	0.15	12.3	0.8
	LVEF	0.97	0.92	1.02	0.27
CMR	LVEDV indexed	1.02	0.96	1.08	0.58
	LV mass indexed	1	0.92	1.08	0.92
	LVEF	1.07	1	1.15	0.056
	RVEDV indexed	0.99	0.93	1.05	0.72
	RVEF	1.11	0.92	1.34	0.29

BAME, Black and Minority Ethnic group; CKD, chronic kidney disease; PAD, peripheral arterial disease; MI, myocardial infarction; renin angiotensin aldosterone system inhibitors; MRA, mineralocorticoid receptor antagonist; LBBB, left bundle branch block; eGFR, estimate glomerular filtration rate; CRP, C-reactive protein; ESR, erythrocyte sedimentation rate; TSH, thyroid stimulating hormone; ULN, upper limit of normal; NT-proBNP, N-terminal pro-brain natriuretic peptide; LVEDD, left ventricular end-diastolic dimension; LVEDV, left ventricular end diastolic volume; IVSDd, Interventricular septal diameter in diastole; LVSD, left ventricular systolic dysfunction; LVEF, left ventricular ejection fraction; LV, left ventricle; RVEDV, right ventricular end diastolic volume; RVEF, right ventricular ejection fraction.

## Discussion

We report one of the largest, single-centre observational analyses of patients hospitalised with AM. We used the ESC position statement on myocarditis and 2018 Lake Louise Criteria to identify nearly 200 patients with CMR-confirmed AM. Importantly, this is the first study of AM in an ethnically diverse population and the first to examine, in detail, sex differences in CMR-proven AM. There are three main findings from our analysis. First, the overall prognosis of CMR-proven AM is benign, with a 3-year event-free survival of 96%. Furthermore, adverse outcomes were virtually exclusively confined to patients with arrhythmia presentations (11% at 3 years of follow-up). Second, there are no major differences in baseline characteristics between patients with different ethnicities. Third, while outcomes are similar for men and women with myocarditis, women have a distinct phenotype, including prior autoimmune disease, more comorbidities, and more breathlessness presentations.

The presentation of AM is highly heterogeneous. Consequently, its epidemiology is incompletely understood (5, 6, 23, 26). Historically, the diagnosis of AM relied on histology obtained from EMB. However, EMB is invasive and not universally available, significantly limiting the widespread applicability of this technique and likely underestimating the real prevalence of the disease (23). Furthermore, most data on the natural history of AM comes from highly selected, EMB-confirmed cases. With increasing availability of CMR, the diagnosis of AM has become more accessible (5, 24, 26, 27). As a result, there is increasing research interest in the use of CMR, rather than EMB, to confirm AM cases. The increased diagnostic yield achieved with CMR has allowed a more detailed and accurate characterisation of patients across the spectrum of AM presentations.

In our cohort, the most common presentation was with chest pain, accounting for three-quarters of cases, with smaller numbers presenting with breathlessness or malignant arrhythmias. These findings are very similar to those of Ammirati et al., who described the clinical course of 443 patients with AM in Italy (15). In their cohort, 73% of patients presented with uncomplicated AM, defined as AM with preserved LVEF and no significant arrhythmia, of whom 97% presented with chest pain. Furthermore, the 5-year incidence of heart transplantation or death in their study was low (5.2%) (15). They found that adverse events were virtually completely confined to patients with a breathlessness or arrhythmia presentations, and that outcomes in patients presenting with chest pain without significant LVSD were good (5-year incidence of death or heart transplantation of 18 vs. 0.3%, respectively). Our study supports these findings in a different healthcare system, time period, and diverse patient population.

Interestingly, we observed a worse prognosis in the arrhythmia compared to breathlessness group. This is contrary to a previous study that described worse clinical outcomes in patients with heart failure presentations than observed in our cohort (11). However, this study used heart transplantation in EMB-proven AM as an endpoint, which is likely to select for sicker patients and may partially account for the different findings. Furthermore, our breathlessness cohort was not limited to patients with LVSD. Our analysis provides a more heterogeneous and contemporary population, and therefore reflects the current prognosis across the spectrum of AM. Arrhythmia patients were older (53 ± 14 years) and, interestingly, less pro-inflammatory than breathless patients. Arrhythmia patients also had larger LV dimensions at presentation, which might suggest AM is a “second hit” in these patients (28, 29).

A higher incidence of AM among men is consistent across studies (4). Previous epidemiological studies have found women with AM to be older, less frequently present with ST elevation, and have no differences in LVEF or all-cause mortality (30, 31). This is consistent with our findings. Mirna et al. also observed lower presenting CRP in women. Animal models of autoimmune myocarditis have suggested attenuation of the immune response in female rats (32). In particular, female rats display preserved LVEF, higher anti-inflammatory Arg1^+^ macrophages, and a lack of increase in pro-inflammatory modulators (such as TLR4, IL6, IL1β, and iNOS). However, we did not observe differences in CRP, instead finding more neutrophils despite significantly lower troponin. We also observed more autoimmune disorders and immunosuppression in women, suggesting complex interactions between AM, sex and immunity. Finally, this is the first study to specifically examine the role of ethnicity in AM. Ethnic variation has been implicated in several cardiovascular conditions, including heart failure, and response to treatment (33–35). We did not observe any major difference between White and BAME patients, and ethnicity was not associated with outcome in univariable analyses.

The use of advanced or immunosuppressive therapy in hospital was low, and mostly confined to patients presenting with breathlessness. In the total population there was limited use of inotropes (9%), IVIG (1%), steroids (7%), and/or RRT (6%). Use of these therapies was slightly lower than described elsewhere, likely reflecting differences in local practices but also reflecting the benign nature of most AM cases diagnosed using CMR. In our study, a lower proportion of patients with breathlessness were managed on cardiology wards. This was not associated with worse outcomes in our study, despite being well known that patients with acute HF have worse outcomes when managed on general medical wards (36–38).

The adverse event rate in our study is significantly lower than that seen in many previous studies. Most of these studies were performed prior to the widespread availability of CMR and used EMB to confirm AM. It is therefore likely that this predominantly reflects differences in study design and patient selection, leading to lower estimates of mortality. Most studies with ≥90% of cases confirmed by CMR indicate 100% survival with follow-up up to 5 years (14–16, 19, 20, 39, 40), with the most conservative estimate being 95.7% survival at 4.7 years of follow-up (17). This is significantly higher than EMB-selected cohorts, that have reported mortality as high as 56% at 4.3 years (41). The variables with the strongest association with adverse events were clinical presentation, and markers of organ damage or compromised haemodynamics. Most of these variables are available at initial presentation and may help identify which patients might need more advanced support or observation.

### Limitations

As a retrospective study of prospectively collected data we recognise several important limitations. We attempted to capture all patients admitted with AM by performing keyword searches of electronic health records and death certificates. However, some patients with AM may have been missed, potentially leading to selection bias. We included patients admitted to two hospitals with AM. The patient populations served by these hospitals may not reflect the general population in the UK or other countries. Furthermore, clinical practice may differ from other national and international centres, which may limit the generalisability of our results. Although all cardiac follow-up that occurred in our institution was captured, it was possible that some endpoints were missed. Patients that moved abroad and subsequently died would have been missed, as would patients that had ICDs implanted at another centre. Finally, unstable patients who could not undergo CMR may have been missed. EMB was not routinely performed and post-mortem analysis was not available, so we were unable to assess aetiology. Despite these limitations, our findings are very similar to those of a comparable multicentre study in terms of clinical presentation and outcomes (15). Finally, although ours is a relatively large study by the standards of AM, our cohort was small with a low number of events. We could therefore not perform multivariable analysis and our study power was limited in identifying variables with a less strong association with adverse events. The use of surrogate endpoints in future AM studies (such as change in EF) may provide sufficient power to identify factors that are independently associated with outcome, especially in male and female subgroups. In addition, studies that examine inflammation in patients with AM would be welcomed.

## Conclusion

Acute myocarditis has highly heterogeneous clinical presentations. In our analysis, two thirds of patients with myocarditis present with chest pain and we identified several differences between male and female patients. Overall, AM is associated with a benign prognosis, especially for chest pain presentations. Those presenting with life-threatening arrhythmias are at higher risk of adverse events.

## Data availability statement

The raw data supporting the conclusions of this article will be made available by the authors, without undue reservation.

## Ethics statement

Ethical review and approval was not required for the study on human participants in accordance with the local legislation and institutional requirements. Written informed consent for participation was not required for this study in accordance with the national legislation and the institutional requirements.

## Author contributions

AC, TM, PS, and DB: design of the study. AC, PB, RR, MA-A, AD, EF, AJ, AS, JH, BC, SB, AK, MS, SR, AB, IR, SP, DS, PS, and DB: data collection. AC, PS, and DB: data analysis and writing the manuscript. MG, AMS, TM, PS, and DB: supervision. All authors critically revised the manuscript.
